# Identification and profiling of *Trichinella spiralis* circulating antigens and proteins in sera of mice with trichinellosis

**DOI:** 10.1371/journal.pone.0265013

**Published:** 2022-03-10

**Authors:** Charin Thawornkuno, Kathyleen Nogrado, Poom Adisakwattana, Tipparat Thiangtrongjit, Onrapak Reamtong

**Affiliations:** 1 Department of Molecular Tropical Medicine and Genetics, Faculty of Tropical Medicine, Mahidol University, Bangkok, Thailand; 2 Department of Helminthology, Faculty of Tropical Medicine, Mahidol University, Bangkok, Thailand; University of Bari, ITALY

## Abstract

Trichinellosis is a zoonotic disease caused by the ingestion of the *Trichinella* nematode. With a worldwide incidence of approximately 10,000 cases per year, *Trichinella spiralis* is responsible for most human infections. There are no specific signs or symptoms of this parasitic infection. Muscle biopsy is the gold diagnostic standard for trichinellosis, but the technique is invasive and unable to detect the early stage of infection. Although immunodiagnostics are also available, antibody detection usually occurs after 3 weeks and prolonged up to 19 years after the acute phase. Therefore, additional diagnostic biomarkers must be identified to improve trichinellosis diagnosis. This study aimed to measure concentration changes in mouse serum proteins prior to *T*. *spiralis* infection and 2, 4 and 8 weeks after infection, and to identify *T*. *spiralis* circulating proteins and antigens using mass spectrometry-based proteomics. Mouse muscle-related proteins including inter-alpha-trypsin inhibitor heavy chain H2, a protein involved in the response to muscle tissue damage, were up-regulated in mouse sera during the *T*. *spiralis* larvae invasion. Additionally, 33 circulatory parasite proteins were identified in infected mouse sera. Notably, *T*. *spiralis* long-chain fatty acid transport protein 1 could be detected in the early stage of infection and peroxidasin-like protein was identified 2, 4 and 8 weeks after infection. Seventeen *T*. *spiralis* circulating antigens were detected in mouse immune complexes, with PX domain protein being found 2, 4 and 8 weeks after infection. Because peroxidasin-like protein and PX domain protein were detected at all post-infection time points, sequence alignments of these proteins were performed, which showed they are conserved among *Trichinella* spp. and have less similarity to the human and murine sequences. Integrative analysis of *T*. *spiralis* biomarkers throughout the course of infection may reveal additional diagnostic targets to improve early diagnosis of trichinellosis.

## Introduction

Trichinellosis also called trichinosis is a zoonotic disease caused by the ingestion of the intracellular nematode, *Trichinella* spp. via the consumption of undercooked or raw meat usually pork and has a worldwide incidence of 10,000 infections per year [1]. Following ingestion of encysted larvae, first-stage larvae are released in the stomach by the action of pepsin and hydrochloric acid. The new born larvae (NBL) then invade the small intestine, where they develop into adults and mate. NBL can enter the lymphatic circulation and then the blood, where they can reach oxygen-rich skeletal muscles, myocardium and brain. To date, there have been no reports of human-to-human transmission. In addition to being found worldwide in wild animals, *Trichinella* is endemic in pig breeding populations in eastern Europe, Russia, China, South Asia and South America [2]. At least 13 Trichinella species/genotypes have been identified [3]. The species responsible for most human Trichinellosis infections is *Trichinella spiralis*, although *T*. *nativa*, *T*. *nelsoni*, *T*. *britovi*, *T*. *pseudospiralis*, *T*. *murelli* and *T*. *papuae* [4, 5] can also be involved.

Human trichinellosis infections can be classified as acute or chronic. An acute-stage infection normally begins with non-specific clinical symptoms such as headache, fever, fever with chills, and gastrointestinal symptoms. Symptoms usually start 1 week after ingestion and fever can persist for 1 to 3 weeks, depending on infection dose and severity of disease. Chronic-stage infection usually occurs 3 to 4 weeks after ingestion and is characterized by encephalitis and secondary infections such as bronchopneumonia or sepsis. Neurological complications rarely occur [6]. Since there are no specific signs or symptoms for human trichinellosis, diagnosis is based on three main criteria, namely epidemiological investigation, clinical findings and laboratory tests (i.e., muscle biopsy or a serological tests such as ELISA and western blot) [6]. Muscle biopsy is the gold standard diagnostic technique, but it is invasive and unable to detect early infection [6]. Immunodiagnostics are also available; however, antibodies are usually detected 3 to 5 weeks after infection [7]. In addition, antibody levels do not correlate with the severity of the clinical course [8] and have been detected up to 19 years after the end of the acute phase [9]. Therefore, trichinellosis diagnosis need to be improved. Potential biomarkers for diagnosis of infectious diseases include changes in host protein levels, detection of pathogen proteins in host specimens and the presence of pathogen antigens that trigger a host immune response. All of these biomarkers can be measured using mass spectrometry-based proteomics.

Proteomics is a high-throughput technology that can provide a global picture of protein composition in various types of biological specimens. It has been used for the identification of potential diagnostic biomarkers, drug target proteins and vaccine candidates. In particular for T. spiralis, surface proteins of muscle and of the infective intestinal larvae [10], as well as excretory–secretory proteins of L1 stage larvae were revealed using proteomic approaches [11]. Moreover, adult worm excretory–secretory proteins recognized by patient sera were reported [12]. Quantitative proteomics was used to study the molting mechanism using *T*. *spiralis* muscle larvae at the encapsulated stage and intestinal infective larvae at the molting stage. The identification of *T*. *spiralis* molting-related proteins aids in the development of vaccines and novel treatment [13]. Comparative protein expression profile of *T*. *spiralis* muscle larvae in response to albendazole sulfoxide (ABZSO), the main intermediary metabolic product of ABZ was studied. The findings help to discover the mechanism of ABZSO actions on *T*. *spiralis*. [14]. Immunoreactive proteins of the *T*. *spiralis* muscle larvae and adult stage recognized by experimentally infected pig sera were also revealed by proteomics [15]. According to several proteomics studies, these findings are useful for diagnosis, vaccine and drug development, and better understanding the molecular biology of trichinellosis. However, only a few studies have attempted to generate diagnostic biomarkers that might be beneficial for early diagnosis [16, 17]. Therefore, this study aimed to quantify changes in the concentration of mouse serum proteins during *T*. *spiralis* infection. The parasite circulating proteins at 2, 4 and 8 weeks after infection were also explored. In addition, *T*. *spiralis* antigens in mouse immune complex at 2, 4 and 8 weeks after infection were indicated. Integrative analysis of the time-course information could provide additional diagnostic biomarker datasets to improve early diagnosis of trichinellosis.

## Materials and methods

### 1. Preparation of infected mouse sera

All animal procedures were approved by the Faculty of Tropical Medicine Animal Care and Use Committee (FTM-ACUC), Mahidol University (approval number 015/2021). The laboratory strains of *T*. *spiralis* used in this study were maintained in the Animal Care Unit, Faculty of Tropical Medicine, Mahidol University. Eight-week-old female ICR mice (3 mice) were fed with 100 larvae by oral gavage. Nembutal® (Pentobarbital) was severed as an anesthesia. Mice infected with 100 larvae did not show any sign of illness during 8 weeks after infection. Blood was collected from the submandibular vein before infection and 2, 4 and 8 weeks after infection, and approximately 200 μL of blood was allowed to clot by leaving the collection tube undisturbed for 30 min at room temperature. Sera were then harvested by centrifugation at 2,000 ×g for 10 min at 4°C and stored at −20°C until use. Three biological replicates were obtained. After experiment, euthanasia method for mice was CO_2_-compressed carbon dioxide gas in cylinders.

### 2. Serum protein separation

To identify changes in concentration of mouse serum proteins and in *T*. *spiralis* circulating proteins post infection, a 30 μg sample of mouse serum from each time point was separated using 12% SDS-PAGE. Protein bands were visualized by staining with Coomassie Blue G, excised from the gel and cut into 12 small pieces for in-gel digestion.

### 3. *T*. *spiralis* circulatory antigen extraction

To capture host antibody interactions with the circulatory antigens of *T*. *spiralis*, coimmunoprecipitation of immune complexes in mouse serum was performed using protein A/G magnetic beads (Pierce™ LSKMAGKP02 kit; Millipore Corporation, USA). Following manufacturer’s instructions, a 50 μL (0.5 mg) portion of beads was added to 150 μL of binding/wash buffer, gently mixed and the supernatant discarded. Pooled sera (10 μL) collected at each time point were diluted with 490 μL of binding/wash buffer, then added to the beads. After mixing at room temperature for 1 h, the supernatant was discarded and the beads were washed twice with 500 μL of binding/wash buffer. Following addition of 50 μL of elution buffer, the samples were incubated 10 min at room temperature with occasional mixing, then the supernatant was collected after centrifugation. Next, a 20 μL portion of each eluate was separated using 12% SDS-PAGE. Protein bands were stained using Coomassie Blue G, then each lane of the gel was sliced horizontally into equal 11 pieces and subjected to in-gel tryptic digestion.

### 4. In-gel digestion

Coomassie dye was removed by incubating gels in 25 mM ammonium bicarbonate buffer containing 50% acetonitrile. Proteins were reduced with 4 mM dithiothreitol (Sigma-Aldrich, St. Louis, MO, USA) in 50 mM ammonium bicarbonate buffer, then alkylated with 250 mM iodoacetamide (Sigma-Aldrich, St. Louis, MO, USA) and dehydrated with 100% acetonitrile. After removing the supernatant, proteins were digested overnight with 10 ng trypsin (Sigma-Aldrich, St. Louis, MO, USA) dissolved in 200 μL of 50 mM ammonium bicarbonate buffer containing 5% acetonitrile. The peptides were extracted by adding 200 μL of acetonitrile and incubating for 20 min. The supernatant containing the peptides was transferred to a new tube and dried using a centrifugal vacuum concentrator, then dissolved in 0.1% v/v formic acid.

### 5. Mass spectrometric analysis

Each peptide mixture was injected into a nano liquid chromatography system (Dionex UltiMate 3000, Surrey, UK). Peptide separation was performed at flow rate of 300 nL/min using an Acclaim PepMap RSLC nanoviper analytical column (75 μm × 15 cm, C18, 2 μm particle size, 100 Å pore size (Thermo Scientific, Waltham, MA). The mobile phase contained 0.1% formic acid in water (A) and 80% acetonitrile in 0.1% formic acid (B). Elution was performed using a 30 min gradient from 4% to 50% B, and the eluted peptides were fed into a micrOTOF-Q mass spectrometer (Bruker Daltonics, Bremen, Germany). Mass spectrometry (MS) and tandem mass spectrometry (MS/MS) data covered m/z ranges of 400–2000 and 50–1500, respectively. A Mascot generic file (.mgf) was generated using the DataAnalysis 3.4 software (Bruker Daltonics). Mascot Daemon version 2.3.2 (Matrix Science, London, UK) was used to merge the.mgf files and identify the proteins.

To identify the circulating proteins and circulating antigens in mouse immune complexes, the *T*. *spiralis* sequences stored in the National Center for Biotechnology Information (NCBI) database were used. The Mascot search allowed up to 1 missed cleavage and a peptide tolerance of 0.8 Da for both MS and MS/MS spectra. Methionine oxidation and cysteine carbamidomethylation were identified as variable modifications. The protein abundance was determined semi-quantitatively using the exponentially modified protein abundance index. Data were visualized using a volcano plot and statistical significance (t-test, *p* < 0.05) was calculated using the Perseus software platform (https://maxquant.net/perseus/). The STRING database (https://string-db.org/) was used to analyze protein–protein interactions.

### 6. Bioinformatic analysis

For sequence alignment, all sequences were retrieved from the non-redundant protein sequence database of the NCBI. The peroxidasin-like protein were KRY43094.1 (*T*. *spiralis*), KRY60541.1 (*T*. *britovi*), KRZ61524.1 (*T*. *nativa*), KRY73684.1 (*T*. *pseudospiralis*), KRZ80717.1 (*T*. *papuae*), XP_006515269.1 (*M*. *musculus*) and XP_011508698.1 (*H*. *sapiens*). The PX domain protein were XP_003372131.1 (*T*. *spiralis*), KRY49378.1 (*T*. *britovi*), KRZ58398.1 (*T*. *nativa*), KRY87520.1 (*T*. *pseudospiralis*), KRZ73230.1 (*T*. *papuae*), EDL26533.1 (*M*. *musculus*) and AAD27836.1 (*H*. *sapiens*). All sequence alignments and identity calculations were performed using the Clustal Omega software.

The SignalP 5.0 server (http://www.cbs.dtu.dk/services/SignalP/index.php) was used to predict the presence of signal peptides in the identified proteins, with a SignalP score greater than 0.9 being defined as “Yes” [18]. The SecretomeP 2.0 server (http://www.cbs.dtu.dk/services/SecretomeP) was used for prediction of non-classical protein secretion, with a SecretomeP score greater than 0.6 in mammalian proteins being defined as “Yes” [19].

## Results

### 1. Proteomic analysis of *T*. *spiralis*-infected mouse sera

**Fig 1:**
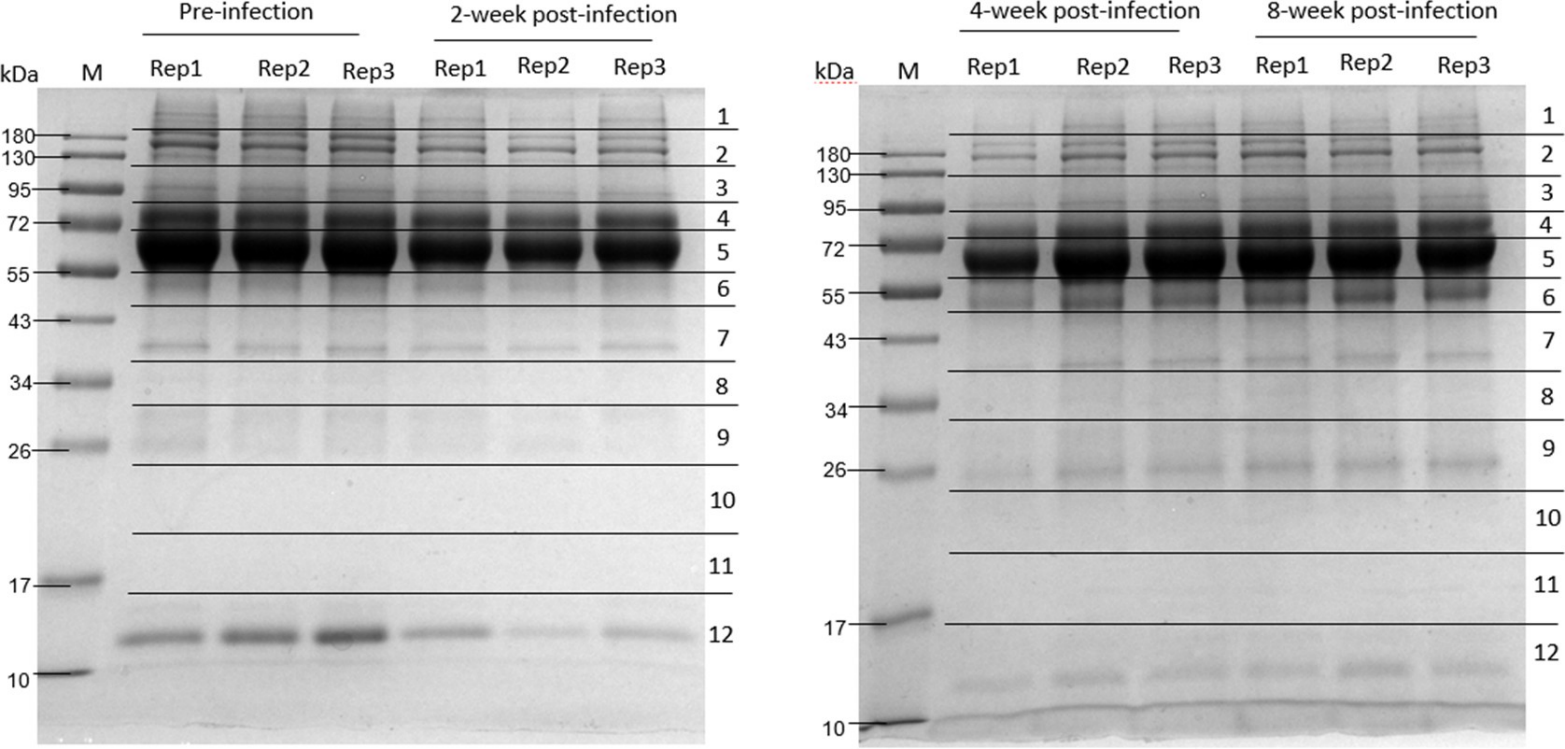
SDS-PAGE of *T*. *spiralis* uninfected and infected mouse sera. Lanes (from left to right): marker, uninfected, 2, 4 and 8 weeks post infection. The 11 horizontal sections show the regions excised for MS analyses.

SDS-PAGE analysis revealed changes in mouse serum protein concentrations before and 2, 4 and 8 weeks after *T*. *spiralis* infection (Fig 1 and S1 Fig), and proteomic analysis respectively identified 2,200, 2,420, 2,589 and 2,670 term proteins (S1 Table).

**Fig 2:**
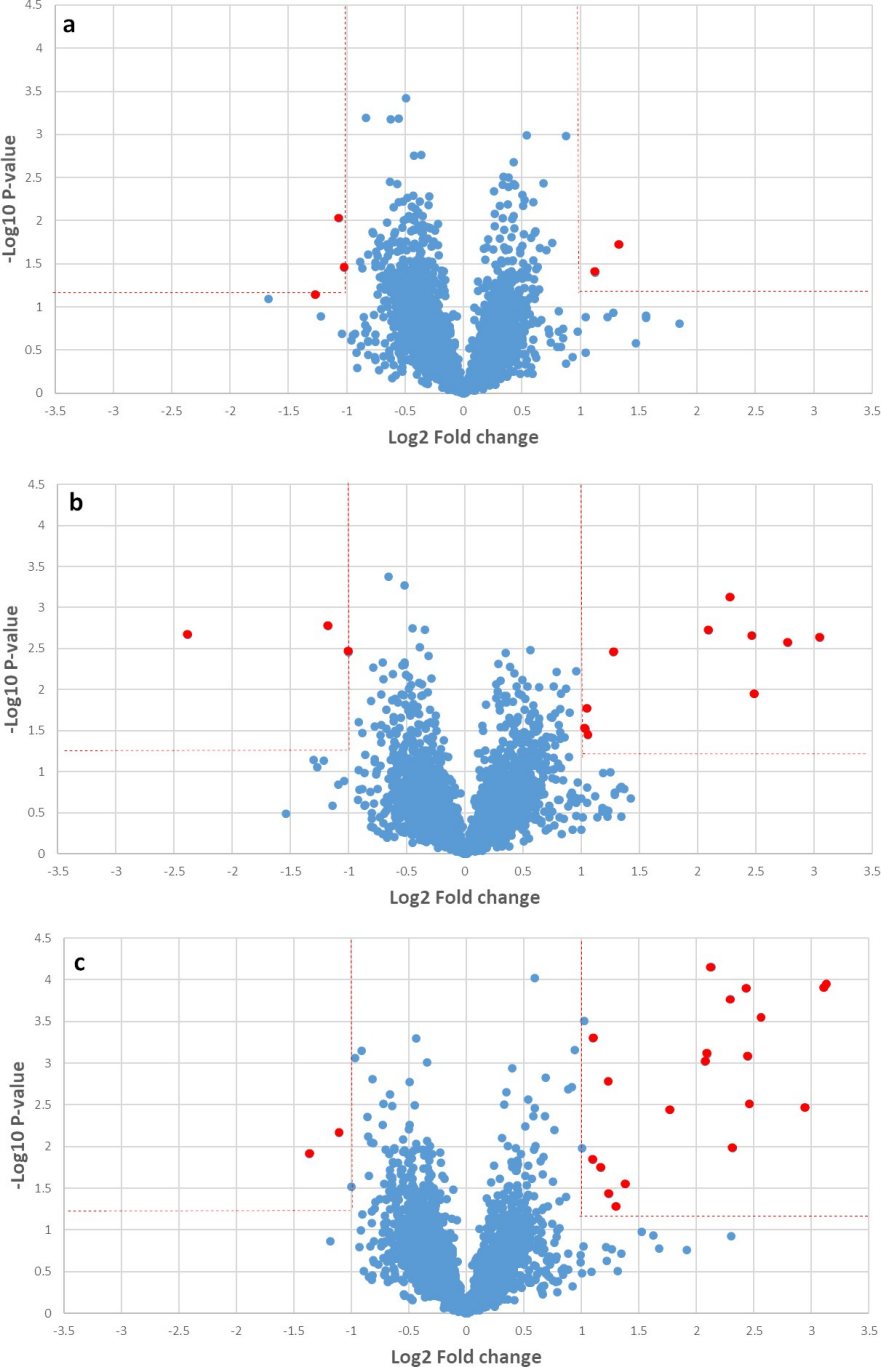
Volcano plots comparing the expression of mouse proteins (blue dots) in mouse sera infected with *T*. *spiralis* 2 (a), 4 (b) and 8 weeks (c) post infection with those in uninfected sera. Statistically significant differential expression of proteins (red dots) is defined as a minimum 2-fold change relative to the uninfected condition (level of magnitude, vertical lines) and *p* < 0.05 (level of statistical significance, horizontal line).

**Fig 3:**
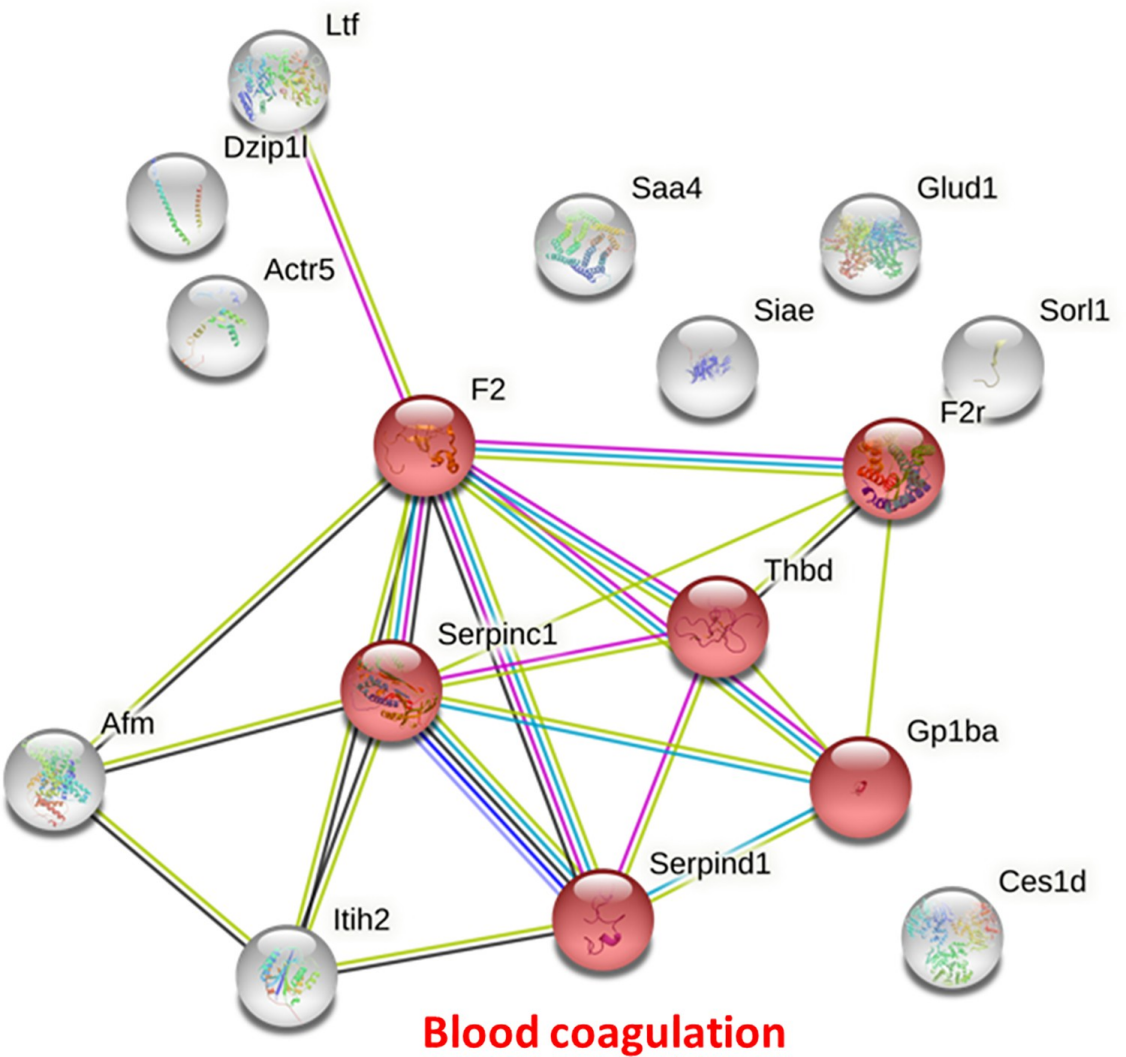
Protein–protein interactions of up-regulated mouse serum proteins 8 weeks post *T*. *spiralis* infection. Proteins identified by mass spectrometry were analyzed for their interactions using the STRING database. Red nodes represent proteins in the blood coagulation pathway which was predicted as the significant altered network after *T*. *spiralis* infection. The abbreviations for each protein are F2: Prothrombin, F2r: Thrombin receptor, Thbd: Thrombomodulin, Serpinc1: Serine/cysteine peptidase inhibitor clade c member 1, Serpind1: Serine/cysteine peptidase inhibitor clade d member 1, Gp1ba: Platelet glycoprotein.

The up-regulation or down-regulation in the proteins expression levels were determined by comparison with the data from uninfected sera. There were significant differences in mouse serum protein concentrations at all three post-infection time points (Fig 2 and Table 1), there being more up-regulated proteins than down-regulated proteins. Two weeks after infection, 2 out of 5 mouse serum proteins were upregulated (mouse complement factor H [FH] and UDP-N-acetylhexosamine pyrophosphorylase). Four weeks after infection, 10 proteins were upregulated (6 immunoglobulins, together with ATP-sensitive inward rectifier potassium channel 11, actin-related protein 5, alkaline phosphatase, tissue-nonspecific isozyme and junctophilin-3) and three were down-regulated (E3 ubiquitin-protein ligase RNF34, eukaryotic initiation factor 4A-III and protein AMBP). Eight weeks after infection, 20 mouse serum proteins were up-regulated (various immunoglobulins, apolipoprotein A-II, serum amyloid A-4 protein, afamin, inter-alpha-trypsin inhibitor heavy chain H2, carboxylesterase 1D, lactotransferrin, sialate O-acetylesterase, prothrombin, zinc finger protein DZIP1L and actin-related protein 5) and two were down-regulated (sortilin-related receptor and mitochondrial glutamate dehydrogenase 1). The protein–protein interactions of differential mouse serum proteins at 2, 4, 8 weeks post infection were analyzed using the STRING database. None of significant molecular processes were found at 2 and 4 weeks post infection. Only up-regulated mouse serum proteins at 8 weeks post infection demonstrated blood coagulation was the major differential pathway after *T*. *spiralis* infection (Fig 3). The alteration of proteins involved in blood coagulation was also found in 2 and 4 weeks after infection, namely complement factor H and protein AMBP.

**Table 1:** Differential mouse serum protein at 2, 4 and 8 weeks after *T*. *spiralis* infection. The up-regulation or down-regulation of the mouse serum proteins expression levels were determined by comparison with uninfected sera using LC-MS/MS. The UniProt protein database was used with *Mus musculus* (house mouse) set as the taxonomy filter. Only the significant differences (p-value ≤ 0.05) were presented in this table.

Accession	Protein	Score	M.W.	No. of peptide	% cov	pI	Fold change	P-value
**2 weeks**								
P06909	Complement factor H	579	139047	23	24.7	6.67	2.51	0.0189
Q91YN5	UDP-N-acetylhexosamine pyrophosphorylase	43	58572	5	10.2	6.04	2.18	0.0401
O08677	Kininogen-1	100	73056	7	16.3	6.05	-2.03	0.0348
Q00623	Apolipoprotein A-I	781	30597	19	51.1	5.51	-2.10	0.0094
Q07456	Protein AMBP	171	39004	8	36.1	5.96	-2.41	0.0723
**4 weeks**								
P01670	Ig kappa chain V-III region PC 6684	154	12032	3	31.5	7.98	8.27	0.0023
P01659	Ig kappa chain V-III region TEPC 124	169	12331	3	31.3	10.02	6.83	0.0027
P01654	Ig kappa chain V-III region PC 2880/PC 1229	200	11973	4	45.9	5.18	5.61	0.0113
P01674	Ig kappa chain V-III region PC 2154	109	11692	2	27.8	5.83	5.53	0.0022
Q8VEB3	Macrophage immunometabolism regulator	50	23116	4	24.2	9.51	4.85	0.0008
P01680	Ig kappa chain V-IV region S107B	139	13825	7	50.4	8.62	4.26	0.0019
Q61743	ATP-sensitive inward rectifier potassium channel 11	37	43534	3	10.3	8.44	2.42	0.0035
Q80US4	Actin-related protein 5	60	67802	3	5.1	5.11	2.08	0.0359
P09242	Alkaline phosphatase, tissue-nonspecific isozyme	35	57478	3	8.4	6.52	2.07	0.0168
Q9ET77	Junctophilin-3	45	81180	7	16.4	9.28	2.05	0.0304
Q99KR6	E3 ubiquitin-protein ligase RNF34	40	42003	8	30.3	4.7	-2.01	0.0035
Q91VC3	Eukaryotic initiation factor 4A-III	57	46810	6	26.8	6.3	-2.26	0.0017
Q07456	Protein AMBP	171	39004	8	36.1	5.96	-5.22	0.0021
**8 weeks**								
P01670	Ig kappa chain V-III region PC 6684	154	12032	3	31.5	7.98	8.70	0.0001
P01635	Ig kappa chain V-V region K2	222	12573	4	44.3	8.5	8.62	0.0001
P03977	Ig kappa chain V-III region 50S10.1	194	12035	4	66.7	4.9	7.67	0.0034
P01659	Ig kappa chain V-III region TEPC 124	169	12331	3	31.3	10.02	5.90	0.0003
P01654	Ig kappa chain V-III region PC 2880/PC 1229	200	11973	4	45.9	5.18	5.51	0.0031
P01674	Ig kappa chain V-III region PC 2154	109	11692	2	27.8	5.83	5.45	0.0008
P09813	Apolipoprotein A-II	81	11302	4	59.8	6.56	5.40	0.0001
P01872	Immunoglobulin heavy constant mu	817	49940	16	42.7	6.56	4.96	0.0104
P01639	Ig kappa chain V-V region MOPC 41	152	14302	4	43.1	5.32	4.91	0.0002
P31532	Serum amyloid A-4 protein	59	15078	1	15.4	9.3	4.37	0.0001
P01636	Ig kappa chain V-V region MOPC 149	135	12023	2	25.9	6.92	4.26	0.0008
P01680	Ig kappa chain V-IV region S107B	139	13825	7	50.4	8.62	4.22	0.0010
O89020	Afamin	184	69334	9	19.9	5.54	3.41	0.0036
Q61703	Inter-alpha-trypsin inhibitor heavy chain H2	199	105861	11	17.1	6.82	2.60	0.0278
Q8VCT4	Carboxylesterase 1D	128	61749	6	11.5	6.17	2.36	0.0370
P08071	Lactotransferrin	84	77788	8	17.5	8.86	2.35	0.0017
P70665	Sialate O-acetylesterase	42	60736	4	14.6	6.32	2.24	0.0179
P19221	Prothrombin	133	70224	8	16.5	6.04	2.14	0.0144
Q499E4	Zinc finger protein DZIP1L	58	87540	9	17.6	8.43	2.03	0.0003
Q80US4	Actin-related protein 5	60	67802	3	5.1	5.11	2.01	0.0105
O88307	Sortilin-related receptor	91	246928	17	11.6	5.3	-2.15	0.0068
P26443	Glutamate dehydrogenase 1, mitochondrial	60	61298	5	11.8	8.05	-2.58	0.0124

### 2. Determination of *T*. *spiralis* circulating proteins in infected mouse sera

Thirty-three *T*. *spiralis* circulating proteins were identified using proteomics (Table 2 and S2 Table). Among them, 6, 13 and 22 proteins were determined 2, 4 and 8 weeks after infection, respectively. No *Trichinella* proteins were detected in uninfected mouse sera. *T*. *spiralis* peroxidasin-like protein, peroxidasin (KRY43095.1) and WD repeat-containing protein 44 (KRY28736.1) were identified with the highest scores in the infected sera 2 weeks post infection. *T*. *spiralis* muscle M-line assembly protein unc-89 (KRY36723.1), hypothetical protein T01_4395 (KRY33215.1) and putative protein tag-76 (KRY38162.1) were discovered with the highest confidence 4 weeks after infection. Eight weeks post infection, *T*. *spiralis* muscle M-line assembly protein unc-89, intron-binding protein aquarius (KRY36956.1) and WD repeat-containing protein 44 (KRY28737.1) were found in the infected mouse sera with the highest confidence. The SignalP and SecretomeP servers predicted that 11 out of 33 proteins were possible secretory proteins, such as acetylcholine receptor subunit alpha-like 1, long-chain fatty acid transport protein 1 and peroxidasin-like protein. Interestingly, peroxidasin-like protein was observed at all three post-infection time points (Table 2). In addition, four *T*. *spiralis* proteins were found both 4 and 8 weeks after infection, namely hypothetical protein T01_16145 (KRY36525.1), conserved hypothetical protein (XP_003366234.1), calcium-dependent secretion activator 1 (XP_003375206.1) and protein CLEC16A (KRY38014.1). The peroxidasin-like protein may be a good biomarker for *T*. *spiralis* diagnosis.

**Table 2:** Identification of *T*. *spiralis* circulatory proteins in mouse infected sera at 2, 4 and 8 weeks after *T*. *spiralis* infection using LC-MS/MS. The NCBI database was used with *T*. *spiralis* set as the taxonomy filter. The SignalP score greater than 0.9 and SecretomeP score greater than 0.6 were defined as classical and non-classical protein secretion, respectively.

Accession	Protein	Score	M.W. (Dalton)	No. of peptide	% coverage	pI	SignalP (>0.9)	SecretomeP (>0.6)
**2 weeks**								
KRY30768.1	Calumenin-A, partial	33	36512	1	6.6	4.32	Yes	Yes
KRY36094.1	hypothetical protein T01_3198	38	38606	1	3.9	9.3	No	No
KRY37099.1	Long-chain fatty acid transport protein 1	52	77353	3	5.7	8.73	No	Yes
KRY28736.1	WD repeat-containing protein 44	82	151552	6	4.9	6.75	No	No
KRY43094.1	Peroxidasin-like protein	105	165950	8	8.1	8.22	Yes	No
KRY43095.1	Peroxidasin	105	135808	8	9.6	6.97	Yes	No
**4 weeks**								
KRY34747.1	Trafficking protein particle complex subunit 5	29	21203	1	4.8	9.33	No	No
KRY36525.1	hypothetical protein T01_16145	30	12971	1	8.4	7.68	No	No
XP_003366234.1	conserved hypothetical protein	30	13988	1	5.7	9.43	No	No
KRY32894.1	hypothetical protein T01_14269	32	13152	1	6.3	9.39	No	Yes
KRX98062.1	hypothetical protein T01_10307	61	6864	3	57.6	9.43	No	Yes
XP_003381646.1	putative transcription factor TFIIB repeat-containing domain protein	70	150245	5	6	6.39	No	No
XP_003381315.1	enoyl-coA hydratase/isomerase family protein	82	27343	6	29.6	9.2	No	No
XP_003375206.1	calcium-dependent secretion activator 1	92	131873	6	6.2	8.57	No	No
KRY38014.1	Protein CLEC16A	92	150023	7	6.4	5.9	No	No
KRY43094.1	Peroxidasin-like protein	97	165950	7	7.3	8.22	Yes	No
KRY38162.1	putative protein tag-76	107	126311	9	12.7	8.89	No	No
KRY33215.1	hypothetical protein T01_4395	141	125540	11	11.9	9.02	No	No
KRY36723.1	Muscle M-line assembly protein unc-89	480	812617	42	8.7	4.96	No	No
**8 weeks**								
KRY36525.1	hypothetical protein T01_16145	29	12971	1	8.4	7.68	No	No
XP_003366234.1	conserved hypothetical protein	41	13988	2	23.6	9.43	No	No
XP_003381745.1	hypothetical protein Tsp_07425	45	14465	2	11	9.4	No	Yes
KRY41817.1	hypothetical protein T01_5853,	49	36142	3	16.6	9.07	No	Yes
XP_003374981.1	autophagy protein 16	58	22226	3	13.8	5.24	No	Yes
KRY37146.1	Coiled-coil domain-containing protein -like	60	73209	4	9.9	7.25	No	No
KRY37099.1	Long-chain fatty acid transport protein 1	71	77353	5	10.4	8.73	No	Yes
KRY38014.1	Protein CLEC16A	79	150023	6	5.4	5.9	No	No
XP_003375206.1	calcium-dependent secretion activator 1	85	131873	5	5	8.57	No	No
XP_003379646.1	putative tetratricopeptide repeat-containing domain protein,	87	90527	5	11	8.86	No	No
KRY40954.1	RNA polymerase-associated protein CTR9 -like protein	87	136006	7	7.8	6.89	No	No
KRY29858.1	Pre-mRNA-splicing factor SYF1	97	106496	6	8.7	5.98	No	No
XP_003374494.1	putative PH domain protein	100	107834	8	8.6	8.96	No	No
KRY38013.1	Protein CLEC16A	109	238739	9	5.9	6.1	No	No
KRY43094.1	Peroxidasin-like protein	115	165950	9	8.5	8.22	Yes	No
KRY38021.1	Protein CLEC16A	117	239233	10	6.4	6.08	No	No
KRY32860.1	Leucine-rich repeat-containing protein let-4	143	256496	12	7.6	7.9	Yes	No
KRY28737.1	WD repeat-containing protein 44	147	120168	11	15	9.44	No	No
XP_003376056.1	secretin receptor	148	188095	11	11.7	7.18	No	No
KRY28736.1	WD repeat-containing protein 44	177	151552	14	12.8	6.75	No	No
KRY36956.1	Intron-binding protein aquarius	346	703602	32	8.8	5.93	No	No
KRY36724.1	Muscle M-line assembly protein unc-89	483	817486	41	9.3	4.96	No	No

### 3. Detection of circulating *T*. *spiralis* antigens in mouse immune complexes

**Fig 4:**
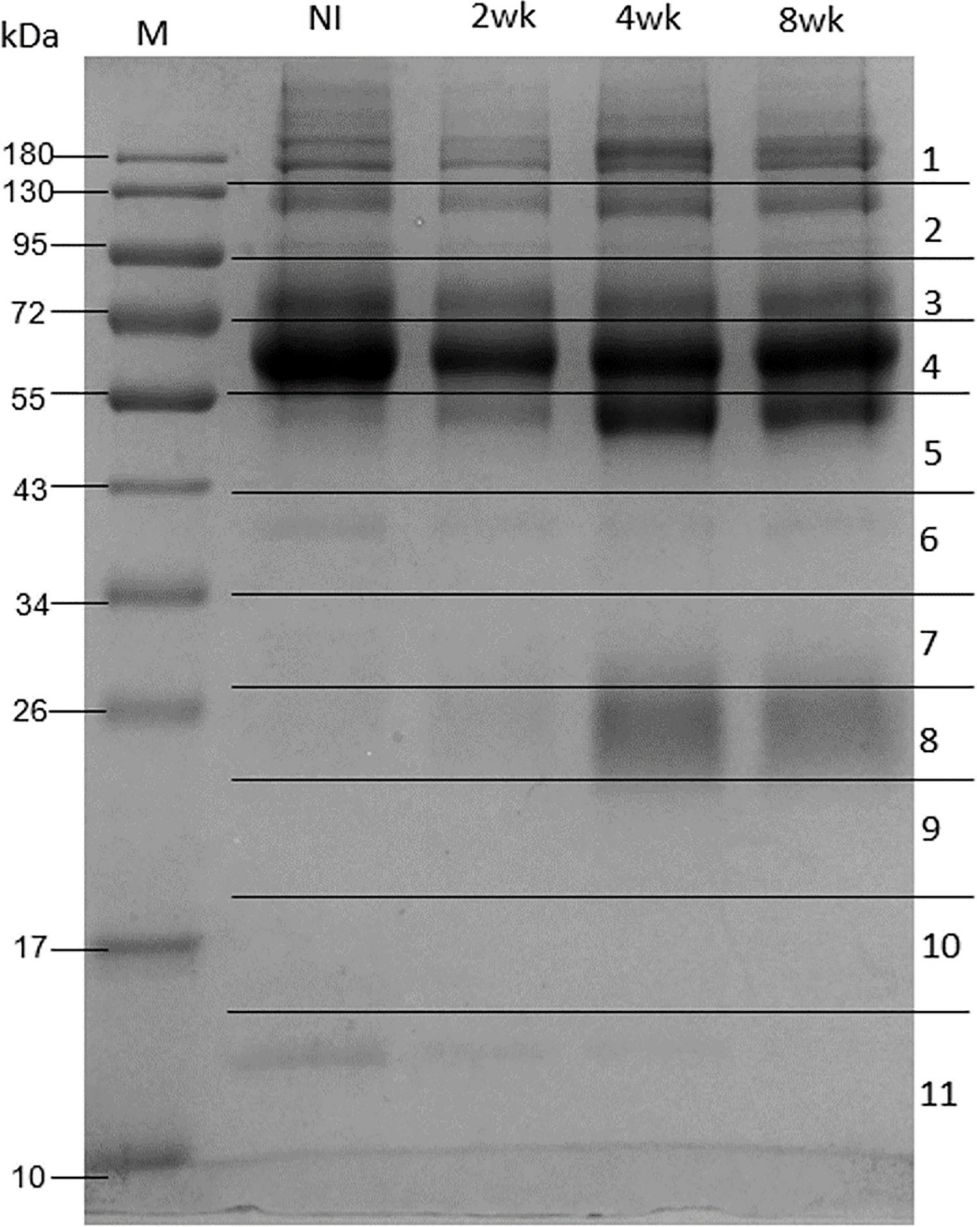
SDS-PAGE of *T*. *spiralis* circulatory antigens from immune complex in *T*. *spiralis* uninfected and infected mouse sera. The immune complexes in *T*. *spiralis* uninfected and infected mouse serum were enriched using protein A/G magnetic bead. and further separated by 12% gel electrophoresis. Lanes (from left to right): marker, uninfected, 2, 4 and 8 weeks post infection. The 11 horizontal sections show the regions excised for MS analyses.

Coomassie-stained SDS-PAGE separations of the immune complexes isolated 4 and 8 weeks after infection revealed increased intensities of protein bands at 26 and 55 kDa compared with those measured in uninfected sera (Fig 4 and S2 Fig). Mass spectrometric analysis of gel no. 5 and no. 8 was performed and identified using UniProt protein database with *Mus musculus* set as the taxonomy filter. The results showed that they were heavy chains (HVM12_MOUSE) and light chains of mouse immunoglobulin G (KV5A7_MOUSE), respectively. Mass spectrometry detected no *Trichinella* spp. proteins in the immune complexes of uninfected mice. Seventeen *T*. *spiralis* proteins were identified in infected mouse immune complexes (Table 3 and S3 Table). Among them, 2, 8 and 12 *T*. *spiralis* proteins were identified 2, 4 and 8 weeks post infection, respectively. Importantly, PX domain protein was identified at all three post-infection time points, and enoyl-coA hydratase/isomerase family protein (XP_003381315.1) and cuticle collagen were identified both 4 and 8 weeks after infection. All of the proteins observed in *T*. *spiralis*-infected mouse immune complexes have also been identified as antigens following infection with other pathogens such as *Trichuris trichiura*, *Hymenolepis diminuta* and *Trichinella pseudospiralis*. In addition, previous immunoproteomic studies have identified hypothetical protein Tsp_10331 (XP_003372574.1), conserved hypothetical protein (XP_003374582.1), putative LNS2 protein (OUC42377.1), hypothetical protein D917_07875 (OUC46244.1), peptidase S1C family (XP_003375617.1), phosphatidylinositol phosphatase PTPRQ (KRY42983.1) and putative zinc finger protein C06E1.8 (KRY37548.1) as *T*. *spiralis* antigens [20, 21].

**Table 3:** Identification of *T*. *spiralis* proteins from infected mouse immune complex at 2, 4 and 8 weeks after *T*. *spiralis* infection. The immune complexes in *T*. *spiralis* uninfected and infected mouse serum were enriched using protein A/G magnetic bead. The *T*. *spiralis* proteins from infected mouse immune complex were identified using LC-MS/MS and the NCBI database was used with *T*. *spiralis* set as the taxonomy filter.

Accession	Protein	Score	M.W.	No. of peptide	% cov	pI	Reported as antigens in other organisms	Ref
**2 weeks**								
KRY41834.1	Acetylcholine receptor subunit alpha-like 1	45	54833	1	1.7	6.28	*Nippostrongylus brasiliensis*	[22]
OUC46688.1	PX domain protein	57	90769	2	2.1	7.19	*Leishmania infantum*	[23]
**4 weeks**								
OUC48320.1	cuticle collagen	48	28324	1	7.5	7.48	*Trichuris trichiura*	[24]
XP_003372574.1	hypothetical protein Tsp_10331	59	24134	2	14.4	7.42	*Trichinella spiralis*	[25]
	putative LNS2 protein	63	94409	3	7.0	7.26	*Trichinella spiralis*	[20]
OUC46244.1	hypothetical protein D917_07875	67	89289	4	7.8	9.78	*Trichinella spiralis*	[20]
KRY41834.1	Acetylcholine receptor subunit alpha-like 1	68	54833	4	13.8	6.28	*Nippostrongylus brasiliensis*	[22]
XP_003381315.1	enoyl-coA hydratase/isomerase family protein	72	27343	4	21.9	9.2	*Hymenolepis diminuta*	[26]
XP_003374582.1	conserved hypothetical protein	73	83428	4	8.0	7.19	*Trichinella spiralis*	[20]
OUC46688.1	PX domain protein	92	90769	5	10.1	7.19	*Leishmania infantum*	[23]
**8 weeks**								
KRY37429.1	hypothetical protein T01_16154	39	14884	1	7.1	9.17	*Brugia malayi*	[27]
OUC48320.1	cuticle collagen	39	28324	1	7.5	7.48	*Trichuris trichiura*	[24]
KRY37548.1	putative zinc finger protein C06E1.8	45	81047	2	2.4	5.71	*Trichinella spiralis*	[20]
KRY34169.1	3-hydroxypropionyl-coenzyme A dehydratase	53	36802	2	6.9	9.42	*Aspergillus fumigatus*	[28]
OUC43754.1	putative tudor domain protein	57	64197	3	9.5	7.84	Human immunodeficiency virus	[29]
OUC49733.1	putative Ion channel	59	55734	3	10.1	9.66	*Gnathostoma spinigerum*	[30]
XP_003375617.1	peptidase, S1C family	62	42594	3	6.2	9.33	*Trichinella spiralis*	[20]
OUC48548.1	Myb-like DNA-binding domain protein	67	77218	4	6.0	8.83	*Trichomonas vaginalis*	[31]
XP_003381315.1	enoyl-coA hydratase/isomerase family protein	77	27343	5	27.9	9.2	*Hymenolepis diminuta*	[26]
XP_003378299.1	hypothetical protein Tsp_06213	81	109804	5	5.0	5.35	*Trichinella pseudospiralis*	[32]
OUC46688.1	PX domain protein	104	90769	4	6.5	7.19	*Leishmania infantum*	[23]
KRY42983.1	Phosphatidylinositol phosphatase PTPRQ	165	525529	14	3.7	7.97	*Trichinella spiralis*	[20]

### 4. Protein sequence alignment of *T*. *spiralis* circulating proteins and antigens

Peroxidasin-like protein and PX domain protein were identified as a *T*. *spiralis* circulating protein and antigen, respectively, 2, 4 and 8 weeks after infection. They may therefore be diagnostic markers of *T*. *spiralis* throughout the course of infection, especially during early stages (at least 2 weeks post infection). Sequence alignments of peroxidasin-like protein and PX domain protein revealed more than 90% similarity among *Trichinella* spp., whereas murine and human sequences of these proteins have less than 50% identity to *Trichinella* spp. (Table 4). This finding indicates peroxidasin-like protein and PX domain protein are possible diagnostic markers for trichinellosis.

**Table 4:** The percent similarity of protein sequence alignment of peroxidasin-like protein (a) and PX domain protein (b) among *Trichinella spp*., *Mus musculus* and *Homo sapiens*. All protein sequences were retrieved from the non-redundant protein sequence database of the NCBI. The sequence alignments and identity calculations were performed using the Clustal Omega software.

**(a)**	
	*T*.*spiralis*
*T*.*spiralis*	100
*T*.*britovi*	97.79
*T*.*nativa*	97.52
*T*.*pseudospiralis*	95.27
*T*.*papuae*	95.86
*M*.*musculus*	35.86
*H*.*sapiens*	29.85
**(b)**	
	*T*.*spiralis*
*T*.*spiralis*	100
*T*.*britovi*	96.93
*T*.*nativa*	96.81
*T*.*pseudospiralis*	88.54
*T*.*papuae*	87.78
*M*.*musculus*	28.25
*H*.*sapiens*	30.19

## Discussion

In this study, proteomic analyses of mouse sera before and after *T*. *spiralis*-infection were performed. After infection, mouse serum proteins were mainly up-regulated. Two weeks post infection, no changes in immunoglobulin protein concentration were detected. However, immunoglobulins were the most up-regulated proteins 4 and 8 weeks after infection, consistent with a previous report that antibody production in human patients is normally detected 2 weeks post infection [33]. Two weeks after *T*. *spiralis* infection, mouse complement FH and UDP-N-acetylhexosamine pyrophosphorylase were up-regulated. FH is a complement control protein belonging to the regulators of complement activation family, which is essential for controlling the alternative complement pathway [34]. During malaria infection, *Plasmodium falciparum* can hijack the host FH on their surface to protect against complement-mediated clearance. The concentration of serum FH in severe malaria is higher than that in uncomplicated malaria, supporting its roles in immune evasion and host clinical manifestations [35]. There is no information on the function of FH in *Trichinella* infection; however, increasing FH concentration during the early stages of infection may protect larvae against complement killing while traveling through the bloodstream prior to infecting striated muscle. UDP-N-acetylhexosamine pyrophosphorylase is involved in first step of UDP-N-acetyl-alpha-D-glucosamine biosynthesis. There is little information about the role of this protein in *Trichinella* infection and other infectious diseases.

Mouse kininogen-1 and apolipoprotein A-I were down-regulated during *T*. *spiralis* infection. Kininogen-1 is a precursor protein for high-molecular-weight kininogen, low-molecular-weight kininogen and bradykinin, which are involved in blood coagulation and inflammation regulation [36]. In *Schistosoma mansoni*, calpain appears to proteolytically cleave and inactivate murine kininogen [37]. Schistosomes cleave the host kininogen to prevent blood clotting and inflammation, allowing them to promote movement throughout the host environment. *Trichinella spiralis* may also cleave kininogen to escape the murine immune system, resulting in down-regulation of mouse kininogen. Apolipoprotein A-I is the major component of high-density lipoprotein particles. A comparative proteomic analysis of *T*. *pseudospiralis*-infected pig sera reported the down-regulation of this protein [38]. Therefore, apolipoprotein A-I is down-regulated in both pigs and mice after *Trichinella* infection. Four weeks post infection, several muscle-related mouse proteins to were up-regulated, such as actin-related protein 5, junctophilin-3, ATP-sensitive inward rectifier potassium channel 11 and tissue-nonspecific alkaline phosphatase. Actin-related protein 5 and junctophilin-3 form part of the cellular ultrastructure of skeletal muscle [39], whereas ATP-sensitive inward rectifier potassium channel 11 can form smooth muscle-type channels that elevate muscle strength [40]. The concentration of tissue-nonspecific alkaline phosphatase isozyme increases during invasion and encystment of *T*. *spiralis* in rat skeletal muscle fibers, the enzyme being abundant in the T-tubule network of rat skeletal muscle [41]. Up-regulation of these muscle-related proteins might be involved in the encystment process of *T*. *spiralis* larvae, which were not present 2 weeks post infection (intestinal stage) but appeared later (muscle stage).

Eight weeks after *T*. *spiralis* infection, lactotransferrin and inter-alpha-trypsin inhibitor heavy chain H2 were up-regulated. Lactotransferrin is a glycoprotein involved in the innate immune response to viral [42], bacterial and helminthic infections [43]. The increase in lactotransferrin concentration might therefore be part of the mouse immune defense against *T*. *spiralis*. Inter-alpha-trypsin inhibitor heavy chain H2 is a serum protein that can bind to hyaluronan and contribute to the response to tissue injury [44]. Therefore, up-regulation of inter-alpha-trypsin inhibitor heavy chain H2 be a marker of muscle tissue damage caused by the *T*. *spiralis* larvae invasion.

Based on their changes in mouse serum protein concentrations, alkaline phosphatase, tissue-nonspecific isozyme, inter-alpha-trypsin inhibitor heavy chain H2 and apolipoprotein A-I are potential diagnostic markers for trichinellosis. In the future, these markers must be evaluated in human trichinellosis and linked to clinical manifestations to be of benefit to diagnosis and prognosis, respectively. Analysis of protein–protein interactions of up-regulated mouse serum proteins 8 weeks post *T*. *spiralis* infection indicated that blood coagulation was the major differential pathway. In human trichinellosis, thrombotic complications [45, 46] and disseminated intravascular coagulation in arterioles of numerous organs [47] are observed. As reported by several studies above, trichinellosis might affect host blood coagulation by altering the concentration of clotting proteins.

Antibody detection may be of limited use for trichinellosis diagnosis because antibodies are usually present 3 weeks post infection [7]. Moreover, their concentrations do not correlate with the severity of the clinical course [8] and they may only be found long after the acute phase of infection [9]. This study aimed to identify *T*. *spiralis* circulatory proteins and antigens in mouse blood for further diagnosis of trichinellosis using antigen-based detection. Thirty-three *T*. *spiralis* proteins were identified in infected mouse sera. Among them, peroxidasin-like protein was detected 2, 4 and 8 weeks after infection. These *T*. *spiralis* proteins are predicted to be secretory and represent diagnostic candidates because their protein sequences are conserved among *Trichinella* spp. and less similar to mouse and human proteins. Peroxidasin is an extracellular peroxidase found in the excretory–secretory products of *Hymenolepis diminuta* [48]. It plays an important role in detoxification and responds to oxidative stress. In addition, peroxidasin is a proposed target for anthelmintic drug development [49]. In *T*. *spiralis*, this protein may likewise be involved in detoxification processes against the host immune response and thus be a potential maker for trichinellosis diagnosis. Two weeks after infection, *T*. *spiralis* long-chain fatty acid transport protein 1 was detected in infected mouse sera and predicted to be a secretory protein. In nematodes, such lipid binding proteins transport lipids in aqueous compartments and play roles in host–parasite interaction. Long-chain fatty acid transport protein 1 is also found in *T*. *papuae* [50] and may be a useful early diagnostic marker of *T*. *spiralis*. After 4 and 8 weeks’ infection, *T*. *spiralis* muscle M-line assembly protein unc-89 was detected in infected mouse sera. This structural protein is involved in assembly and organization of sarcomere myofilaments and required for normal muscle function in *Caenorhabditis elegans* [51] and *Dirofilaria immitis* [52]. Similarly, this protein may also play a role in the *T*. *spiralis* muscle function and movement. In summary, the identification of *T*. *spiralis* circulating proteins provided several additional diagnostic candidates such as long-chain fatty acid transport protein 1 (for 2 weeks after infection), muscle M-line assembly protein unc-89 (for 4 and 8 weeks after infection) and peroxidasin-like protein (for 2, 4, and 8 weeks after infection. Monoclonal or monospecific antibodies against these candidates must be developed and screened to determine their diagnostic power.

Various methods have been developed to detect circulating antigens of *T*. *spiralis*, including counter-immunoelectrophoresis, immunoradiometric assays, Dot blots, and ELISAs. These methods rely on antigen-antibody interaction. However, none of them could be used to identify antigens from immune complex since most of them detecting free antibody in the host serum. The immune complex information is useful not only as a guide to possible parasite diagnostic and vaccine candidates, but it also provides another potential mechanism of parasite immune evasion, which could lead to a new strategy to treat the parasitic disease [27]. In this study, *T*. *spiralis* antigens in the mouse immune complex were isolated and separated 2, 4 and 8 weeks after infection. Electrophoretic separations of the complexes revealed that the protein bands at 26 and 55 kDa were more intense 4 and 8 weeks after infection compared with those obtained from samples of uninfected mice. These bands correspond to the two 50 kDa heavy chains and two 23.5 kDa light chains of mouse immunoglobulin G, consistent with the fact that the antibody response to trichinellosis usually begins 3 weeks after infection [7]. Seventeen *T*. *spiralis* proteins were identified in infected mouse immune complexes. PX domain protein was found 2, 4 and 8 weeks after *T*. *spiralis* infection. This protein usually plays roles in maintaining normal neuronal excitability and synaptic transmission. It is found in secretions of *Leishmania infantum* promastigotes and reacts to infected dog sera [23]. PX domain protein is therefore a putative target for the diagnosis and treatment of canine visceral leishmaniasis and the development of vaccines against the disease. Enoyl-coA hydratase/isomerase family protein and cuticle collagen were identified both 4 and 8 weeks after infection. The former degrades fatty acids by beta-oxidation [53] and was identified among the surface-associated proteins of *Hymenolepis diminuta* in sera of infected mice [26]. Molting is a critical step in parasitic worm growth and development because it allows the parasites to expand their body size and adapt to their surroundings. *T*. *spiralis* cuticle collagen is up-regulated in muscle larvae at the encapsulated stage compared with its concentration in intestinal infective larvae [13]. *Trichuris trichiura* cuticle collagen from egg extract was identified as an antigen reacting with sera of infected African green monkeys [24]. Identification herein of PX domain protein, enoyl-coA hydratase/isomerase family protein and cuticle collagen as *T*. *spiralis* circulating antigens provides new targets for development of antigen- and antibody-based trichinellosis diagnostics. According to the advantages of circulating parasitic antigen detection, it is one of the most accurate diagnostic method for distinguishing between the active or past infection. It could also be used for evaluation of the chemotherapy efficiency. The findings of our current study could expand the list of known *T*. *spiralis* immunogens and mouse serum biomarkers, which could be useful for further diagnosis and vaccine development. However, there are several limitations which need to be further explored for accomplishing the early trichinellosis diagnosis development. A sandwich ELISA based on IgY and IgM monoclonal antibody against excretory-secretory antigens of *T*. *spiralis* muscle larvae was developed. Based on IgM capturing, the circulating antigens in infected mouse sera were detected at 4 days post infection and reached a peak in heavily infected mice at 10 days post infection [54]. In addition, the *T*. *spiralis* infective larvae undergo four molts within the first 30 hours post infection [55]. Therefore, identification of circulating proteins and antigens in this parasite stage development before 2 weeks after infection as well as indication of antigens in IgM immune complex instead of IgG could add more information for the early diagnosis development. Moreover, antigen identified from infected mice and humans were different [56]. Candidate antigens identified in mouse models might not be suitable in humans. Translation of information from mouse models to human patients needs to be done carefully. The identification of circulating proteins and antigens either in human patients and other hosts could also be useful for early diagnosis in different hosts.

## Conclusion

Integrative information of the alteration of mouse serum proteins and *T*. *spiralis* circulating proteins and antigens at 2, 4, and 8 weeks after infection with *T*. *spiralis* may yield additional diagnostic biomarker datasets to aid in trichinellosis early diagnosis. These discoveries are important for trichinellosis diagnosis, vaccination and treatment development, and a better knowledge of the disease’s molecular biology.

## Supporting information

S1 Table Identification of mouse serum proteins. (XLSX)

S2 Table Identification of *T*. *spiralis* circulating proteins. (XLSX)

S3 Table Identification of *T*. *spiralis* circulating antigens in mouse immune complexes. (XLSX)

S1 Fig Uncropped SDS-PAGE of uninfected and *T*. *spiralis* infected mouse sera. (TIF)

S2 Fig Uncropped SDS-PAGE of *T*. *spiralis* circulatory antigens from immune complex in uninfected and infected mouse sera. (TIF)
