# Supplementary figures and images for: Identification and profiling of Trichinella spiralis circulating antigens and proteins in sera of mice with trichinellosis
Source: PLoS One. 2022 Mar 10;17(3):e0265013. doi: 10.1371/journal.pone.0265013 (PMC8912135; doi:10.1371/journal.pone.0265013)

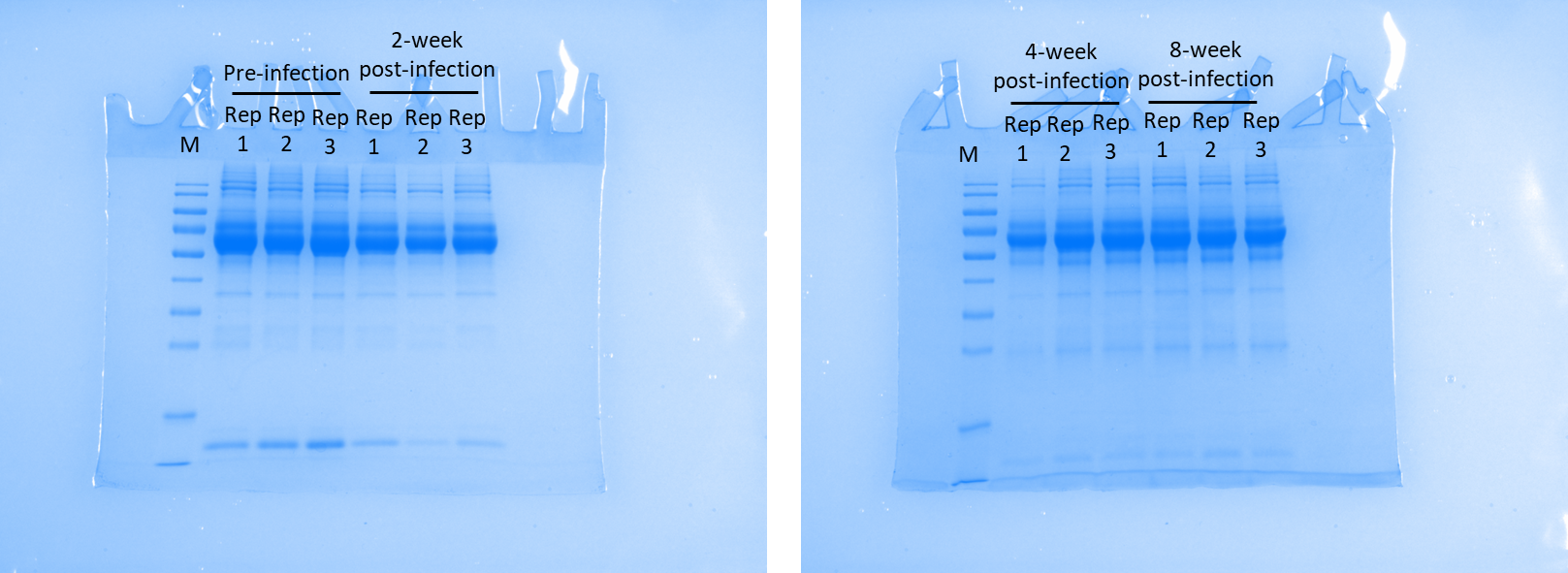

Supplement: S1 Fig — (TIF) [file pone.0265013.s004.tif]

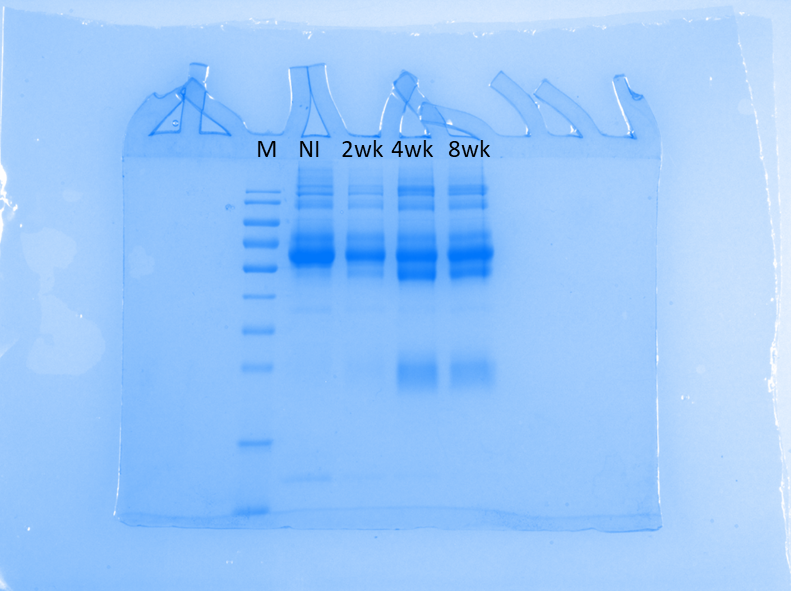

Supplement: S2 Fig — (TIF) [file pone.0265013.s005.tif]
